# Clinical Significance of ErbB Receptor Family in Urothelial Carcinoma of the Bladder: A Systematic Review and Meta-Analysis

**DOI:** 10.1155/2012/181964

**Published:** 2012-09-09

**Authors:** Yuh-Shyan Tsai, Hong-Lin Cheng, Tzong-Shin Tzai, Nan-Haw Chow

**Affiliations:** ^1^Department of Urology, National Cheng Kung University Hospital, Tainan 70403, Taiwan; ^2^Department of Urology, College of Medicine, National Cheng Kung University, Tainan 70403, Taiwan; ^3^Department of Pathology, College of Medicine, National Cheng Kung University Hospital, Tainan 70403, Taiwan

## Abstract

The prognostic importance of examining ErbB receptor family expression in human bladder cancer remains uncertain. Using published evidence, we examined the clinical value and the updated results of clinical trials targeting ErbB receptor family members. Twenty-seven articles from 65 references related to ErbB receptor expression assessment in bladder cancer were reviewed. The estimates included the association significance, hazard ratios, and 95% confidence intervals (CIs) from actuarial curves and survival analyses. A meta-analysis was done on those reports using univariate log-rank tests or a Cox-regression model. The methods of analysis and study subjects chosen varied widely among studies. The overall risks of disease progression for patients with EGFR or ErbB2 overexpression were 4.5 (95% CI: 2.5–8.4) and 1.1 (95% CI: 0.6–1.9), and the risks of mortality were 3.0 (95% CI: 1.6–5.9) and 1.1 (95% CI: 1.0–1.2), respectively. However, the significance of coexpression patterns of the ErbB receptor family remains controversial. None of six clinical trials yielded convincing results for blockading ErbB receptor signaling in urothelial carcinoma. The results of this analysis suggest that assessing co-expression patterns of the ErbB family may provide better prognostic information for bladder cancer patients.

## 1. Introduction

One characteristic of bladder cancer is its variable patient prognosis. About 70% of superficial (Ta and T1) tumors recur, and 10–20% of them become invasive [1]. Tumors that are invasive at primary diagnosis carry a high risk of progression despite radical cystectomy and other auxiliary treatments. Conventional prognostic factors, such as tumor stage, grade, size, and multifocality, do not accurately predict the clinical outcome for some patients. Therefore, extensive efforts have been made to identify biomarkers for predicting disease progression, response to treatment, and chance of long-term survival. Currently, it is recommended that patients with bladder cancer have regular urinary cytology, cystoscopy, and imaging studies at followup [2].

The ErbB receptor family (also known as the epidermal growth factor receptor (EGFR) family) is a major class of receptor tyrosine kinase (RTK) protooncogenes. They are important in many cell regulatory processes, such as proliferation, migration, adhesion, and, potentially, cellular transformation, including urothelial carcinogenesis. The ErbB family consists of 4 members: ErbB1 (also called EGFR and HER1), ErbB2 (c-*erb*B-2 and HER2/*neu*), ErbB3 (HER3), and ErbB4 (HER4). Dimerization by binding two monomers is the regulatory mechanism for activating RTKs [3]. In some cases, the formation of heterodimeric complexes allows interaction and crosstalk between different receptors of the same subfamily, and the ErbB receptor family is the best example of homo- and heterodimerization *in vivo* [4, 5]. Therefore, clarifying the clinical significance of ErbB expression may provide important molecular targets for cancer therapy.

EGFR signaling regulates biological processes important for the pathogenesis of human cancers, including lung cancer, breast cancer, and prostate cancer [6]. In practice, therapy that targets EGFR gene mutations in primary tumors has extended the theme of targeted cancer therapies [7]. In breast cancer, HER2 amplification status is a pivotal biomarker in predicting response to chemotherapy [8], and a humanized anti-HER2 monoclonal antibody (trastuzumab) improved the survival of HER2-positive breast cancer patients [9]. The prognostic significance of ErbB receptor signaling has tissue-specific relevance. For example, EGFR/HER2-MAPK axis is important in human breast cancer while the kinase activity of the HER2/ErbB3 axis plays a major role in the DNA binding and androgen receptor stability in prostate cancer [10]. Moreover, the EGFR inhibitor gefitinib is ineffective in treating hormone-refractory prostate cancer, a result questioning the significance of the EGFR/HER2 axis in the molecular pathogenesis of prostate cancer [11].

To establish the clinical relevance of ErbB receptor family members in bladder cancer, we systematically reviewed the papers published in the past two decades on ErbB receptor family expression, either one of the members or the coexpression patterns, and their impact on patient prognosis. Our objectives were to confirm the significance of ErbB receptor expression in predicting recurrence, progression, and mortality in patients with bladder cancer, to identify factors that might affect the prognostic evaluation of ErbB receptors, and to conduct a meta-analysis of available estimates. In addition, we assessed the potential sources of heterogeneity underlying the conflicting results, and incorporated quantitative methods to analyze the data. The updated results of clinical trials targeting ErbB receptor signaling were also reviewed.

## 2. Materials and Methods

### 2.1. Search Strategy and Selection Criteria

Original articles published between January 1985 and May 2011 showing prognostic significance of expression or amplification of ErbB receptor members in patients with bladder cancer were systematically reviewed. Using the keywords “EGF”, “EGF-R,” “c-erb-B1,” “c-erb-B2,” “c-erb-B3,” “c-erb-B4,” “neu,” “epidermal growth factor,” and “bladder neoplasms or transitional cell carcinomas -in-humans,” we identified 710 relevant articles in the PubMed database. The number of studies was reduced to 65 by limiting the search to “prognosis” or “survival,” including disease recurrence and progression. Duplicate data, identified in the same cohort by reviewing the interstudy similarities of investigators, source of patients, recruitment period, or inclusion criteria, were excluded from the current analysis. Only the largest series were included in our analysis.

### 2.2. Data Extraction, Handling, and Analyses

Our database was designed to ensure the breadth of relevant data obtained, based on study design, patient outcomes, tumor characteristics, statistical analyses, biological samples, analytical methods (namely, immunohistochemistry [IHC], fluorescence in situ hybridization [FISH], and real-time polymerase chain reaction [RT-PCR]), and the incidence of overexpression or gene amplification of ErbB receptor family members.

Two methods were used to summarize the results. Because most articles provided only the *P* values or statements of whether results were significant (and no other measures of effect), the analyses of recurrence, progression, mortality, progression-free survival, disease-free survival, and resistance to chemotherapy and radiotherapy were based on definitions in the original reports. Briefly, recurrence was defined as a tumor that reappeared in the urinary bladder not at a higher T stage, while disease progression was defined as any tumor with a higher T stage in the local tumor, node, or metastasis. The *P* values (or statements of significance) were extracted from association analyses (*χ* ^2^ test, Fisher's exact test, Student's *t*-test, the Mann-Whitney *U*-test, and logistic regression), and the risk estimates and 95% confidence intervals (CIs) from univariate (Kaplan-Meier curves and the log-rank test) and multivariate survival (Cox regression) analyses. Data are summarized by providing the means of percentages or actual numbers with either the standard deviation (SD) or range. *χ* ^2^ tests and Fisher's exact tests were used, when appropriate, to assess the independence of two categorical variables. Unconditional logistic regression models were used to identify the significance of study characteristics (*P* < 0 · 05).

A meta-analysis using multivariate tests was then done. Wolf's method was used to combine the risk estimates by applying the inverse of variance as the weighting factor. Potential sources of heterogeneity were investigated using graphical methods, such as the Galbraith plot. A heterogeneity test based on the statistics was done in all meta-analyses. The heterogeneity was considered significant when *P* < 0.10. In cases of substantial heterogeneity, random-effect models were used. The extent to which the combined risk estimate was affected by individual study was examined by consecutively omitting every study from the meta-analysis. Metaregression was used to explain the potential heterogeneity from the same characteristics included in the *P* value analysis. The publication bias was investigated using Egger's and Begg's graphical methods. The analyses were done using Comprehensive Meta Analysis Version 2 (Biostat, Englewood, NJ, USA). Significance was set at *P* < 0.05 (two sided).

## 3. Results

### 3.1. Significance of Individual ErbB Receptor Expression

**Figure 1:**
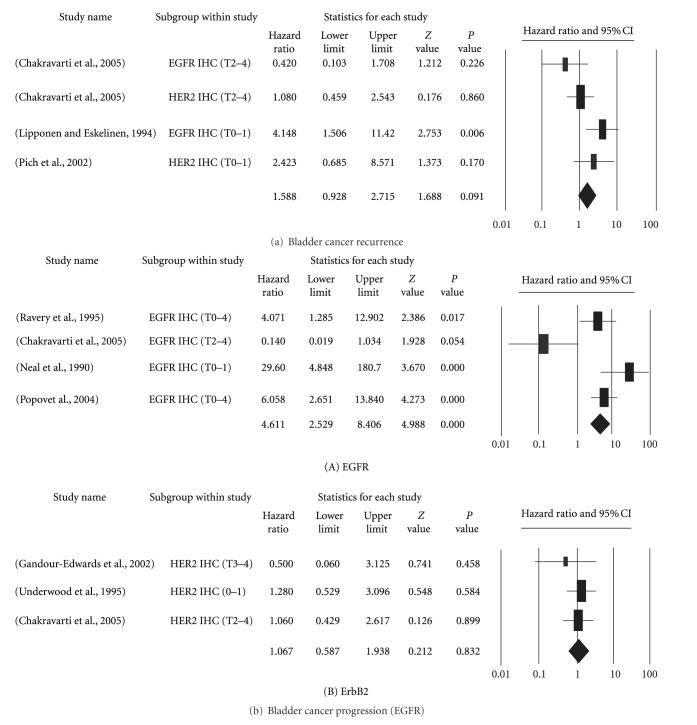
Forest plots of studies on the prognostic significance of EGFR and ErbB2 overexpression in bladder cancer. (a) The effect on recurrence. (b) The effect of EGFR (A) or ErbB2 (B) on progression. Hazard ratios and 95% (confidence intervals) CIs for patients with either EGFR- or ErbB2-positive tumors.

Tumors recurred in 1233 patients in 16 analyses reported in 8 studies (median: 162 patients; range: 52–243) (Table 1) [5, 12–18]. The receptor members analyzed in order of frequency were ErbB2 (7 of 8) and EGFR (6 of 8). Three studies [5, 15, 16] reported significantly positive results. After data processing, only 4 reports were accepted for analysis: 2 for EGFR and 2 for ErbB2 (Figure 1(a)). The distribution of the tumor stages in patients with cancer varied widely. All four studies used IHC to analyze their study samples. Overall, the hazard ratios of tumor recurrence were 1.588 (95% CI: 0.9–2.7).

For cancer progression, 484 patients from 9 analyses were reported in 8 studies (median: 60 patients; range: 21–113) (Table 2) [15, 19–24]. The receptor members analyzed in order of frequency were ErbB2 (5 of 8) and EGFR (4 of 8). Four studies [15, 19, 20, 22] found significantly positive results. After data processing, only 4 reports on EGFR and 3 on ErbB2 were accepted for analysis (Figure 1(b)). The distribution of tumor staging varied widely. All four studies used IHC to analyze their study samples. Overall, the hazard ratios of cancer progression were 4.611 (95% CI: 2.5–8.4) for EGFR and 1.067 (95% CI: 0.59–1.97) and ErbB2 overexpression.

**Figure 2:**
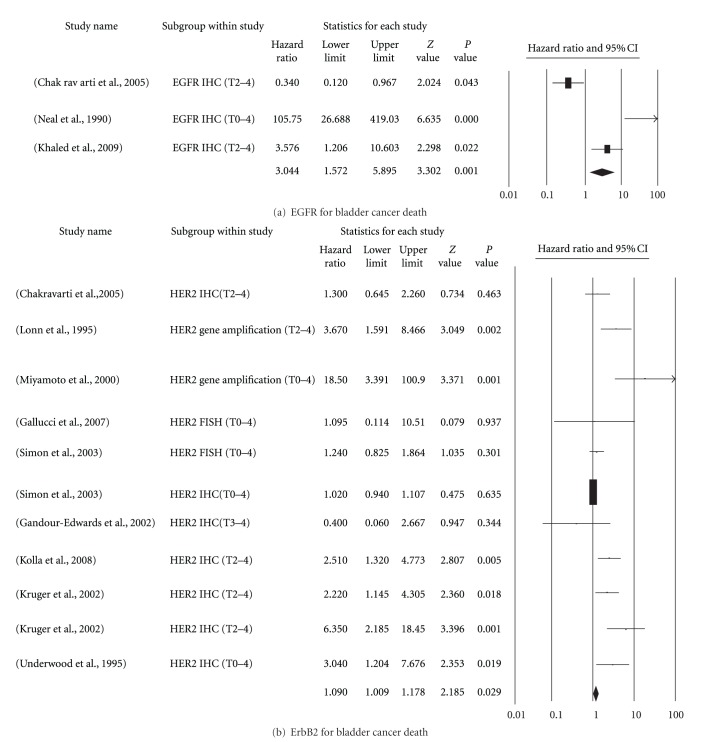
Forest plots of prognostic significance of EGFR and ErbB2 overexpression on bladder cancer death. (a) EGFR. (b) ErbB2. Hazard ratios and 95% (confidence intervals) CIs for patients with either EGFR- or ErbB2-positive tumors.

Cancer-related mortality in 3444 patients from 31 analyses was reported in 20 studies (median: 88 patients, range: 39–1500) (Table 3) [5, 13, 15, 16, 19–27, 34–37]. The receptor members analyzed in order of frequency were ErbB2 (18 of 20) and EGFR 8 of 20). Thirteen studies [5, 13, 15, 16, 21–23, 26, 34–37] reported significantly positive results. After data processing, only 3 reports on EGFR and 10 on ErbB2 were accepted for analysis (Figure 2). The distribution of tumor staging also varied widely. IHC was the most common method of assessment; however, FISH was used in 1 study, and gene copy amplification was used in another. Overall, the hazard ratios of cancer death were 3.044 (95% CI: 1.6–5.9) for EGFR and 1.090 (95% CI: 1.0–1.2) for ErbB2 overexpression.

### 3.2. Significance of the Coexpression Pattern of ErbB Receptors

Three studies [5, 16, 28] investigated the significance of the coexpression patterns of ErbB receptor family members in association with tumor recurrence and patient survival. None of the coexpression patterns were significant in predicting tumor recurrence. In contrast, several coexpression patterns were independent prognostic indicators of bladder cancer death, namely, EGFR-ErbB2, ErbB2-ErbB3 [5], and high EGFR or ErbB2 plus low ErbB3 or ErbB4 [16, 28] (Table 4).

### 3.3. Assessment and Publication Bias

**Figure 3:**
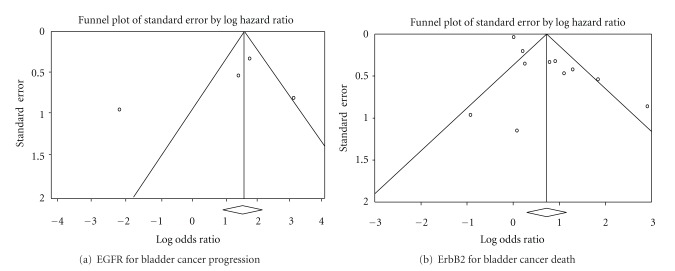
Funnel plots of publication bias for EGFR and ErbB2. (a) No obvious funnel plot asymmetry was found for EGFR because only four studies were analyzed. (b) A substantial funnel plot asymmetry for ErbB2 was found, which suggests the existence of publication bias.

A substantial funnel plot asymmetry suggestive of the publication bias was revealed for ErbB2 (Figure 3(b)). However, no obvious funnel plot asymmetry was found for EGFR, possibly because only 4 studies were analyzed (Figure 3(a)).

### 3.4. Clinical Trials of Targeting ErbB Signaling in Human Bladder Cancer

Until February 2012, there were 6 finished clinical trials targeting ErbB signaling in human bladder cancer (Table 5). Cetuximab is a chimeric (mouse/human) monoclonal antibody for blocking EGFR signaling. A phase II clinical trial [38] of 39 pretreated patients with metastatic urothelial carcinoma demonstrated that combined cetuximab and paclitaxel chemotherapy yielded a better response rate (28.5%) than did cetuximab monotherapy, in which 9 of 11 patients had disease progression at 8 weeks.

Gefitinib is the first selective inhibitor of the EGFR tyrosine kinase domain. An overall response rate of 42.6% (95% CI: 29.2–56.8%) was demonstrated in a phase II trial of 58 patients with metastatic urothelial carcinoma treated with gemcitabine and cisplatin chemotherapy plus gefitinib [39, 40], the median survival time was 15.1 months (95% CI: 11.1–21.7 mo), and time to progression was 7.4 months (95% CI: 5.6–9.2 mo). Twenty-five patients completed the trial without reducing or discontinuing the gefitinib. The authors concluded that this combination therapy was well tolerated and effective in metastatic disease. However, adding gefitinib did not improve the response rate or patient survival. In contrast, a phase II trial of 31 pretreated patients with metastatic urothelial carcinoma [41] reported a response rate of 6.5%, and the authors concluded that gefitinib is ineffective as a second-line agent for metastatic urothelial carcinoma.

Lapatinib is an EGFR and ErbB2 dual tyrosine kinase inhibitor. An objective response rate (ORR) greater than 10% was found in only 1 of 59 patients (1.7%) in a phase II trial using lapatinib as the second-line agent for patients with metastatic urothelial carcinoma [42], and the disease was stabilized in 18 of these patients (31%). The result is basically negative. Interestingly, the clinical advantage (ORR and stable disease) correlated with the EGFR overexpression (*P* = 0.029), and, to some extent, HER-2 overexpression.

Vandetanib is a tyrosine kinase inhibitor for EGFR, vascular endothelial growth factor receptor (VEGFR), and (rearranged during transfection) RET. Choueiri et al. reported that combined vandetanib and docetaxel did not provide more benefit for ORR or patient survival than did docetaxel plus placebo in a double-blind trial of 142 patients with metastatic urothelial carcinoma. The toxicity was also greater in the combination group [43]. Taken together, the outcome of current clinical trials suggests that more investigations are required to identify an appropriate strategy or a more effective agent targeting ErbB signaling in the design of treatment for patients with urothelial carcinoma.

## 4. Discussion

This meta-analysis revealed that estimates of the significance of ErbB receptor family member expression vary substantially between studies. Nonetheless, EGFR overexpression is moderately predictive of progression and mortality in patients with bladder cancer. ErbB2 overexpression is weakly predictive of cancer mortality. Since relatively few studies have examined the implications of ErbB3 and ErbB4, no conclusion can be made at this stage. However, these findings should be interpreted carefully because relatively few studies were eligible for analysis.

It has been more than three decades since the discoveries of EGF and EGFR [44, 45]. Many bladder cancer studies examined the signaling events of this receptor family [16, 46, 47]. Given that human urine contains high concentrations of EGF (20 ng/mg creatinine) [48] and transforming growth factor (TGF)-*α* (0.6 ng/mg creatinine) [49], the interaction of ligands with their cognitive receptors has been thought crucial in the homeostasis of bladder mucosa. This hypothesis is supported by clinical observations that urinary EGF is inversely correlated with the intensity of EGFR expression in primary tumors (*P* = 0.04) [47]. The finding supports the importance of the urinary EGF and urothelial EGFR interaction in the pathogenesis of human bladder cancer.

When ligands bind to the extracellular domains of specific RTKs, tyrosine kinase activity at their intracellular domain is activated [50]. Several regulatory signaling pathways of the ErbB receptor family are important in the proliferation, angiogenesis, migration, and metastasis of human cancer [51]. The conventional paradigm is that aberrant activation of ErbB receptors is generated by the overexpression or mutations of the receptor, and by the autocrine production of ligands [50]. It is now known that signaling output from EGFR is quite complicated. Not only tyrosine-phosphorylated EGFR engages at least six biochemical downstream pathways, but EGFR family RTKs include three other receptors, of which only ErbB4 is autonomous [52]. Other ErbB proteins may be coexpressed and activated in the same cell, resulting in their dimerization with EGFR. The association of EGFR with ErbB2 prevents its downregulation, which reinforces the biological effects of EGFR [53].

ErbB signaling may be terminated or blunted by other mechanisms, such as the dissociation of ligands, which causes dephosphorylation or degradation of the receptors [54, 55]. Alwan et al. hypothesized that proteasomal targeting of ErbB proteins or lysosomal degradation upon ligand-induced endocytosis is involved in EGFR downregulation [56]. Since the ErbB receptor family has many negative feedback mechanisms (e.g., receptor endocytosis, phosphorylation, and ubiquitination/deubiquitination) and crosstalk with other pathways (such as NOTCH pathways, VEGF, and TGF-*β*), components of the EGFR/ErbB network are excellent targets for cancer therapy [57].

Several studies have reported that overexpressed or mutant EGFR family members drive the development of human cancers, including lung [58], breast [59], melanoma [60], prostate [61], and urinary bladder cancer [62]. Alternatively, other aberrations, such as mutant forms of RAF or PI3K, manipulate the downstream signaling in cancer through negative feedback loops [63]. Thus, computational charting of EGFR/ErbB signaling may guide the cell-fate decision. Altogether, it is conceivable to speculate that assessing individual ErbB family receptor expression is insufficient for estimating the biological potential of patients with cancer.

A number of studies have reported the association of EGFR and ErbB2 overexpression with advanced stages of bladder cancer. These phenotypes are thought to predict patient survival, as well as the response to chemotherapy [64, 65]. However, these biomarkers were not included as molecular indicators for patients with bladder cancer [66, 67]. This review describes an authentic inconsistency of the efficacy of ErbB receptor expression in predicting the risk of recurrence, progression, and mortality in patients with bladder cancer. To some extent, this incongruity is consistent with the discouraging results of clinical trials focusing on blockading ErbB receptor signaling [38, 40–43].

Factors responsible for the discrepancies in this meta-analysis include long periods of patient recruitment, a wide range of disease statuses (T0-1, T0–4, T2–4), variability in the immunohistochemical assays used, bias from staining scoring, cutoff points, and subjectivity in interpreting results. There are additional contributing factors, namely, the length of followup, strategies for detecting events of interest, and inconsistency in the inclusion of clinical and pathological factors for multivariate analysis [68]. Not surprisingly, different treatment plans may have different effects on clinical outcome, which might not be considered a confounding factor [69]. The risks calculated in our meta-analysis may be overestimated by reporting biases because hazard ratios and 95% CIs were not described when associations were not significant.

## 5. Conclusions

In conclusion, this meta-analysis has revealed a significant association between ErbB family receptor expression and progression and mortality in patients with bladder cancer even though these findings need to be carefully interpreted. Considering current molecular information, assessing ErbB family coexpression patterns may provide better prognostic information for patients with bladder cancer. Updated clinical trials suggest that more investigations are required to identify effective agents for targeting ErbB signaling of urothelial carcinoma. Systematic reviews and meta-analyses are mandatory before accepting ErbB receptor expression patterns as predictive markers for clinical application.

## Figures and Tables

**Table 1:** Significance of ErbB receptor family as a marker for tumor recurrence.

Study name	Methods	Pts no.	Study subject	Significance
EGFR				
[12]	IHC	230	T0-1	NS
[13]	IHC	73	T2–4	NS
[14]	IHC	141	T0-1	NS
[15]	IHC	52	T0-1	Yes
[16]	IHC	182	T0–4	Yes
[5]	IHC	245	T0–4	NS

ErbB2				
[16]	IHC	182	T0–4	NS
[12]	IHC	230	T0-1	NS
[13]	IHC	55	T2–4	NS
[14]	IHC	141	T0-1	NS
[17]	IHC	62	T0-1	NS
[18]	IHC	248	T0-1	NS
[5]	IHC	245	T0–4	Yes

ErB3				
[16]	IHC	128	T0–4	NS
[5]	IHC	245	T0–4	Yes

ErbB4				
[16]	IHC	124	T0–4	NS

IHC: immunohistochemistry.

**Table 2:** Significance of ErbB receptor family as a marker for cancer progression.

Study name	Methods	Pts no.	Study subject	Significance
EGFR				
[19]	IHC	57	T0–4	Yes
[13]	IHC	73	T2–4	NS
[15]	IHC	52	T0-1	Yes
[20]	IHC	113	T0–4	Yes

ErbB2				
[21]	FISH	62	T0–4	NS
[13]	IHC	55	T2–4	NS
[22]	IHC	39	T3-4	Yes
[23]	IHC	21	T0-1	NS
[24]	IHC	67	T2–4	NS

**Table 3:** Significance of ErbB receptor family as a marker for bladder cancer death.

Study name	Methods	Pts no.	Study subject	Significance
EGFR				
[13]	IHC	73	T2–4	Yes
[25]	IHC	109	T2	NS
[16]	IHC	182	T0–4	NS
[26]	IHC	59	T2–4	Yes
[27]	IHC	141	T2–4	NS
[15]	IHC	52	T0-1	NS
	IHC	101	T0–4	Yes
[26]	RT-PCR	59	T2–4	Yes
[28]	RT-PCR	88	T0–4	NS
[29]	qRT-PCR	73	T0–4	NS

ErbB2				
[30]	Gene amplification	163	T0–4	Yes
[31]	Gene amplification	57	T0–4	Yes
[21]	FISH	62	T0–4	NS
[32]	FISH	1500	T0–4	NS
[13]	IHC	55	T2–4	NS
[22]	IHC	39	T3-4	Yes
[25]	IHC	109	T2	NS
[33]	IHC	80	T2–4	NS
[16]	IHC	184	T0–4	NS
[34]	IHC	90	T2–4	Yes
[35]	IHC	138	T2–4	Yes
[36]	IHC	132	T2–4	Yes
[27]	IHC	141	T2–4	NS
[37]	IHC	88	T0–4	Yes
[32]	IHC	1500	T0–4	NS
[5]	IHC	245	T0–4	Yes
[23]	IHC	89	T0–4	Yes
[28]	RT-PCR	88	T0–4	NS
[29]	RT-PCR	73	T0–4	NS

ErbB3 or ErbB4				
[16]	IHC	128	T0–4	NS
	IHC	124	T0–4	Yes

**Table 4:** Co-expression of ErbB receptor family as a marker for bladder cancer prognosis.

Study name	Methods	Pts no.	Study subject	Significant co-expression pattern for	
				Recurrence	Survival
[5]	IHC	245	T0–4	EGFR-ErbB2-ErbB3*	EGFR-ErbB2
					ErbB2-ErbB3
[16]	IHC	184	T0–4	Not significant	High EGFR + low ErbB4
[28]	RT-PCR	88	T0–4	Not done	High EGFR + low ErbB3 or ErbB4
					High ErbB2 + low ErbB3 or ErbB4

*P = 0.075.

**Table 5:** The update results of published clinical trials targeting EGFR signaling in urothelial carcinoma patients.

Study agents	Pts no.	ORR (%)	Recommendation
EGFR signaling			
Cetuximab + paclitaxel versus cetuximab	39	28.5 versus 18	The combination merits further evaluation [38]
Geftinib + Gemcitabine, cisplatin	58	42.6%	The combination of cisplatin, gemcitabine, and gefitinib is well tolerated, and the addition of gefitinib does not appear to improve response rate or survival [39, 40]
Geftinib	31	6.5	Geftinib (ZD1839) is ineffective as a second-line agent for urothelial carcinoma [41]

EGFR and ErbB2 signaling			
Lapatinib	59	ORR (1.7%) and SD (31%)	A negative result, but clinical benefit (ORR and SD) is correlated with EGFR overexpression (*P* = 0.029), and, to some extent, HER-2 overexpression [42]

EGFR, VEGFR, and RET signaling			
Vandetanib plus docetaxel versus placebo plus docetaxel	142	ORR, 7 versus 11 (*P* = 0.56); PFS, 2.6 m versus 1.6 m (*P* = 0.939); OS, 5.9 m versus 7.0 m (*P* = 0.347)	The addition of vandetanib to docetaxel did not result in a significant improvement in PFS, ORR, or OS [43]

ORR: objective response rate; SD: stable disease, PFS: progression-free survival; OS: overall survival; VEGFR: vascular endothelial growth factor receptor; RET: rearranged during transfection.
